# 2,2,2-Tribromo-*N*-(4-chloro­phen­yl)acetamide

**DOI:** 10.1107/S1600536809032139

**Published:** 2009-08-15

**Authors:** B. Thimme Gowda, Sabine Foro, P. A. Suchetan, Hartmut Fuess

**Affiliations:** a Department of Chemistry, Mangalore University, Mangalagangotri 574 199, Mangalore, India; b Institute of Materials Science, Darmstadt University of Technology, Petersenstrasse 23, D-64287 Darmstadt, Germany

## Abstract

The crystal structure of the title compound, C_8_H_5_Br_3_ClNO, shows both intra­molecular N—H⋯Br and inter­molecular N—H⋯O hydrogen bonding. In the crystal, the mol­ecules are packed into column-like chains in the *c*-axis direction *via* the N—H⋯O hydrogen bonds.

## Related literature

For the preparation of the compound, see: Gowda *et al.* (2003 ▶). For our study of the effect of ring and side-chain substituents on the solid state structures of *N*-aromatic amides, see: Gowda *et al.* (2000 ▶, 2007 ▶, 2009 ▶).
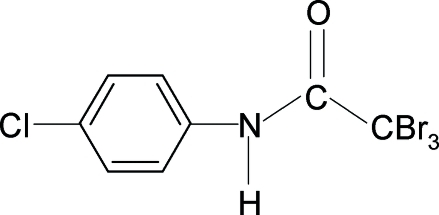


## Experimental

### 

#### Crystal data

C_8_H_5_Br_3_ClNO
*M* *_r_* = 406.31
Orthorhombic, 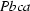

*a* = 9.7332 (8) Å
*b* = 10.2462 (9) Å
*c* = 23.898 (2) Å
*V* = 2383.3 (3) Å^3^
*Z* = 8
Mo *K*α radiation
μ = 10.35 mm^−1^
*T* = 299 K
0.40 × 0.16 × 0.10 mm

#### Data collection

Oxford Diffraction Xcalibur diffractometer with a Sapphire CCD detector
Absorption correction: multi-scan (*CrysAlis RED*; Oxford Diffraction, 2009 ▶) *T*_min_ = 0.104, *T*_max_ = 0.355
5692 measured reflections
2353 independent reflections
1643 reflections with *I* > 2σ(*I*)
*R*_int_ = 0.033

#### Refinement

*R*[*F* ^2^ > 2σ(*F* ^2^)] = 0.080
*wR*(*F* ^2^) = 0.205
*S* = 1.04
2353 reflections
130 parameters
1 restraint
H atoms treated by a mixture of independent and constrained refinement
Δρ_max_ = 2.04 e Å^−3^
Δρ_min_ = −0.95 e Å^−3^

### 

Data collection: *CrysAlis CCD* (Oxford Diffraction, 2009 ▶); cell refinement: *CrysAlis RED* (Oxford Diffraction, 2009 ▶); data reduction: *CrysAlis RED*; program(s) used to solve structure: *SHELXS97* (Sheldrick, 2008 ▶); program(s) used to refine structure: *SHELXL97* (Sheldrick, 2008 ▶); molecular graphics: *PLATON* (Spek, 2009 ▶); software used to prepare material for publication: *SHELXL97*.

## Supplementary Material

Crystal structure: contains datablocks I, global. DOI: 10.1107/S1600536809032139/pk2183sup1.cif

Structure factors: contains datablocks I. DOI: 10.1107/S1600536809032139/pk2183Isup2.hkl

Additional supplementary materials:  crystallographic information; 3D view; checkCIF report

## Figures and Tables

**Table 1:** Hydrogen-bond geometry (Å, °)

*D*—H⋯*A*	*D*—H	H⋯*A*	*D*⋯*A*	*D*—H⋯*A*
N1—H1*N*⋯Br1	0.84 (5)	2.87 (10)	3.197 (8)	105 (8)
N1—H1*N*⋯O1^i^	0.84 (5)	2.21 (5)	3.038 (9)	168 (10)

Symmetry code: (i) 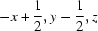.

**Fig. 1.**
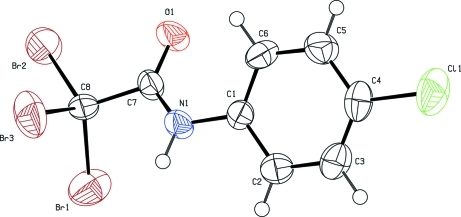
Molecular structure of (I), showing the atom labelling scheme and displacement ellipsoids are drawn at the 50% probability level.

**Fig. 2.**
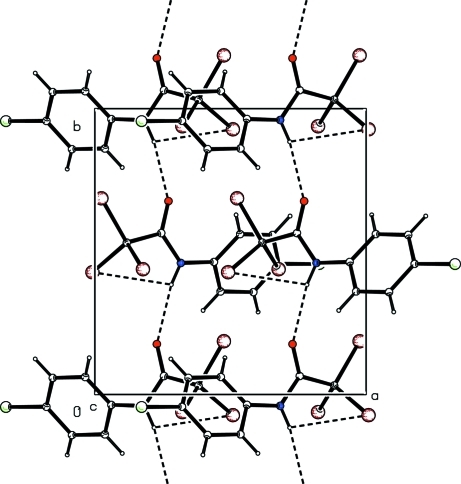
Molecular packing of (I) with hydrogen bonding shown as dashed lines.

## supplementary crystallographic information

### Comment

As part of a study of the effect of the ring and side chain substituents on the solid state structures of *N*-aromatic amides (Gowda *et al.*, 2000, 2007, 2009), the structure of 2,2,2-tribromo-*N*-(4-chlorophenyl)acetamide has been determined (Fig.1).  The conformation of the N—H bond is *anti* to the C=O bond in the side chain, similar to that observed in *N*-(4-chlorophenyl)acetamide (Gowda *et al.*, 2007), 2,2,2-trichloro-*N*-(4-chlorophenyl)acetamide (Gowda *et al.*, 2003), and other amides (Gowda *et al.*, 2009). The structure shows both intramolecular N—H···Br and intermolecular N—H···O H-bonding. The packing diagram of molecules showing the hydrogen bonds N1—H1N···O1 (Table 1) involved in the formation of molecular chains in the direction of the *c*-axis is given in Fig. 2.

### Experimental

The title compound was prepared from 4-chloroaniline, tribromoacetic acid and phosphorylchloride according to the literature method (Gowda *et al.*, 2003). The purity of the compound was checked by determining its melting point. It was further characterized by recording its infrared spectra. Single crystals of the title compound used for X-ray diffraction studies were obtained by a slow evaporation of its solution in petroleum ether at room temperature.

### Refinement

The H atom of the NH group was located in a difference map and later restrained to the distance N—H = 0.86 (5) Å. The other H atoms were positioned with idealized geometry using a riding model [C—H = 0.93 Å]. All H atoms were refined with isotropic displacement parameters (set to 1.2 times of the *U*_eq_ of the parent atom).

The largest residual electron-density features are located in the region of Br3 and Br2. The highest peak is 0.98 Å from Br3 and the deepest hole is 0.50 Å from Br2.

### Crystal data

**Table:**

C_8_H_5_Br_3_ClNO	*F*(000) = 1520
*M**_r_* = 406.31	*D*_x_ = 2.265 Mg m^−^^3^
Orthorhombic, *P**b**c**a*	Mo *K*α radiation, λ = 0.71073 Å
Hall symbol: -P 2ac 2ab	Cell parameters from 2849 reflections
*a* = 9.7332 (8) Å	θ = 2.6–27.8°
*b* = 10.2462 (9) Å	µ = 10.35 mm^−^^1^
*c* = 23.898 (2) Å	*T* = 299 K
*V* = 2383.3 (3) Å^3^	Long needle, colourless
*Z* = 8	0.40 × 0.16 × 0.10 mm

### Data collection

**Table:**

Oxford Diffraction Xcalibur diffractometer with a Sapphire CCD detector	2353 independent reflections
Radiation source: fine-focus sealed tube	1643 reflections with *I* > 2σ(*I*)
graphite	*R*_int_ = 0.033
Rotation method data acquisition using ω and φ scans	θ_max_ = 26.4°, θ_min_ = 2.7°
Absorption correction: multi-scan (*CrysAlis RED*; Oxford Diffraction, 2009)	*h* = −12→8
*T*_min_ = 0.104, *T*_max_ = 0.355	*k* = −12→9
5692 measured reflections	*l* = −29→21

### Refinement

**Table:**

Refinement on *F*^2^	Primary atom site location: structure-invariant direct methods
Least-squares matrix: full	Secondary atom site location: difference Fourier map
*R*[*F*^2^ > 2σ(*F*^2^)] = 0.080	Hydrogen site location: inferred from neighbouring sites
*wR*(*F*^2^) = 0.205	H atoms treated by a mixture of independent and constrained refinement
*S* = 1.04	*w* = 1/[σ^2^(*F*_o_^2^) + (0.0891*P*)^2^ + 22.8289*P*] where *P* = (*F*_o_^2^ + 2*F*_c_^2^)/3
2353 reflections	(Δ/σ)_max_ = 0.005
130 parameters	Δρ_max_ = 2.04 e Å^−^^3^
1 restraint	Δρ_min_ = −0.95 e Å^−^^3^

### Special details

**Table:**

Experimental. CrysAlis RED (Oxford Diffraction, 2009) Empirical absorption correction using spherical harmonics, implemented in SCALE3 ABSPACK scaling algorithm.
Geometry. All e.s.d.'s (except the e.s.d. in the dihedral angle between two l.s. planes) are estimated using the full covariance matrix. The cell e.s.d.'s are taken into account individually in the estimation of e.s.d.'s in distances, angles and torsion angles; correlations between e.s.d.'s in cell parameters are only used when they are defined by crystal symmetry. An approximate (isotropic) treatment of cell e.s.d.'s is used for estimating e.s.d.'s involving l.s. planes.
Refinement. Refinement of *F*^2^ against ALL reflections. The weighted *R*-factor *wR* and goodness of fit *S* are based on *F*^2^, conventional *R*-factors *R* are based on *F*, with *F* set to zero for negative *F*^2^. The threshold expression of *F*^2^ > σ(*F*^2^) is used only for calculating *R*-factors(gt) *etc*. and is not relevant to the choice of reflections for refinement. *R*-factors based on *F*^2^ are statistically about twice as large as those based on *F*, and *R*- factors based on ALL data will be even larger.

### Fractional atomic coordinates and isotropic or equivalent isotropic displacement parameters (Å^2^)

**Table:**

	*x*	*y*	*z*	*U*_iso_*/*U*_eq_	
C1	0.4391 (10)	0.4634 (8)	0.1037 (4)	0.038 (2)	
C2	0.4633 (11)	0.3604 (9)	0.0673 (5)	0.048 (2)	
H2	0.3990	0.2939	0.0633	0.058*	
C3	0.5841 (10)	0.3580 (10)	0.0369 (5)	0.051 (3)	
H3	0.6017	0.2885	0.0129	0.062*	
C4	0.6786 (10)	0.4569 (10)	0.0418 (4)	0.051 (3)	
C5	0.6549 (10)	0.5578 (10)	0.0790 (5)	0.053 (3)	
H5	0.7196	0.6240	0.0829	0.064*	
C6	0.5357 (11)	0.5609 (9)	0.1103 (4)	0.048 (2)	
H6	0.5206	0.6282	0.1357	0.058*	
C7	0.2449 (10)	0.5644 (8)	0.1518 (4)	0.039 (2)	
C8	0.1178 (10)	0.5312 (8)	0.1876 (4)	0.043 (2)	
N1	0.3172 (8)	0.4601 (6)	0.1361 (3)	0.0425 (19)	
H1N	0.283 (10)	0.386 (6)	0.140 (4)	0.051*	
O1	0.2715 (7)	0.6760 (5)	0.1408 (3)	0.0521 (18)	
Cl1	0.8276 (3)	0.4561 (4)	0.00254 (14)	0.0775 (10)	
Br1	−0.00883 (11)	0.42751 (13)	0.14426 (6)	0.0737 (5)	
Br2	0.02425 (15)	0.68618 (11)	0.21207 (7)	0.0817 (5)	
Br3	0.17735 (16)	0.43813 (13)	0.25385 (5)	0.0792 (5)	

### Atomic displacement parameters (Å^2^)

**Table:**

	*U*^11^	*U*^22^	*U*^33^	*U*^12^	*U*^13^	*U*^23^
C1	0.053 (5)	0.028 (4)	0.033 (5)	0.007 (4)	0.005 (4)	0.006 (4)
C2	0.054 (6)	0.036 (5)	0.054 (6)	0.003 (4)	0.005 (5)	−0.001 (5)
C3	0.047 (6)	0.053 (6)	0.054 (6)	0.017 (5)	0.009 (5)	−0.006 (5)
C4	0.040 (5)	0.073 (7)	0.040 (5)	0.011 (5)	0.004 (4)	0.004 (5)
C5	0.040 (5)	0.050 (6)	0.070 (7)	−0.004 (4)	0.008 (5)	−0.006 (5)
C6	0.055 (6)	0.042 (5)	0.046 (6)	0.001 (5)	0.000 (5)	−0.014 (5)
C7	0.042 (4)	0.035 (5)	0.040 (5)	−0.001 (4)	0.009 (4)	0.007 (4)
C8	0.053 (5)	0.023 (4)	0.052 (6)	0.002 (4)	0.011 (5)	−0.005 (4)
N1	0.051 (5)	0.025 (4)	0.052 (5)	0.002 (3)	0.013 (4)	0.007 (3)
O1	0.056 (4)	0.024 (3)	0.077 (5)	0.003 (3)	0.022 (4)	0.003 (3)
Cl1	0.0468 (15)	0.117 (3)	0.069 (2)	0.0073 (16)	0.0172 (14)	−0.0168 (19)
Br1	0.0450 (6)	0.0856 (9)	0.0904 (10)	−0.0022 (6)	−0.0020 (6)	−0.0336 (8)
Br2	0.0900 (9)	0.0439 (6)	0.1112 (12)	0.0043 (6)	0.0532 (8)	−0.0141 (7)
Br3	0.0860 (9)	0.0990 (10)	0.0527 (7)	−0.0108 (7)	0.0088 (7)	0.0255 (7)

### Geometric parameters (Å, °)

**Table:**

C1—C6	1.380 (13)	C5—H5	0.9300
C1—C2	1.389 (13)	C6—H6	0.9300
C1—N1	1.416 (12)	C7—O1	1.202 (10)
C2—C3	1.382 (14)	C7—N1	1.334 (11)
C2—H2	0.9300	C7—C8	1.542 (13)
C3—C4	1.374 (14)	C8—Br2	1.921 (8)
C3—H3	0.9300	C8—Br1	1.929 (10)
C4—C5	1.383 (14)	C8—Br3	1.937 (10)
C4—Cl1	1.727 (10)	N1—H1N	0.84 (5)
C5—C6	1.382 (14)		
			
C6—C1—C2	120.4 (9)	C1—C6—C5	119.5 (9)
C6—C1—N1	121.7 (8)	C1—C6—H6	120.2
C2—C1—N1	117.8 (8)	C5—C6—H6	120.2
C3—C2—C1	119.2 (9)	O1—C7—N1	126.0 (8)
C3—C2—H2	120.4	O1—C7—C8	120.2 (8)
C1—C2—H2	120.4	N1—C7—C8	113.8 (7)
C4—C3—C2	120.8 (9)	C7—C8—Br2	111.5 (6)
C4—C3—H3	119.6	C7—C8—Br1	109.6 (6)
C2—C3—H3	119.6	Br2—C8—Br1	108.4 (5)
C3—C4—C5	119.6 (9)	C7—C8—Br3	108.8 (7)
C3—C4—Cl1	120.8 (8)	Br2—C8—Br3	107.4 (5)
C5—C4—Cl1	119.5 (8)	Br1—C8—Br3	111.0 (4)
C6—C5—C4	120.4 (9)	C7—N1—C1	125.2 (7)
C6—C5—H5	119.8	C7—N1—H1N	119 (7)
C4—C5—H5	119.8	C1—N1—H1N	115 (7)
			
C6—C1—C2—C3	−1.2 (15)	O1—C7—C8—Br2	−2.7 (12)
N1—C1—C2—C3	−177.3 (9)	N1—C7—C8—Br2	176.9 (7)
C1—C2—C3—C4	−1.1 (15)	O1—C7—C8—Br1	117.4 (9)
C2—C3—C4—C5	2.3 (16)	N1—C7—C8—Br1	−63.0 (10)
C2—C3—C4—Cl1	−178.3 (8)	O1—C7—C8—Br3	−121.0 (9)
C3—C4—C5—C6	−1.3 (16)	N1—C7—C8—Br3	58.6 (9)
Cl1—C4—C5—C6	179.3 (8)	O1—C7—N1—C1	0.5 (16)
C2—C1—C6—C5	2.2 (15)	C8—C7—N1—C1	−179.1 (9)
N1—C1—C6—C5	178.1 (9)	C6—C1—N1—C7	36.9 (14)
C4—C5—C6—C1	−1.0 (16)	C2—C1—N1—C7	−147.1 (10)

### Hydrogen-bond geometry (Å, °)

**Table:**

*D*—H···*A*	*D*—H	H···*A*	*D*···*A*	*D*—H···*A*
N1—H1N···Br1	0.84 (5)	2.87 (10)	3.197 (8)	105 (8)
N1—H1N···O1^i^	0.84 (5)	2.21 (5)	3.038 (9)	168 (10)

Symmetry codes: (i) −*x*+1/2, *y*−1/2, *z*.
